# Editorial: Hot Topics of Debate on Turner Syndrome: Growth, Puberty, Cardiovascular Risks, Fertility and Psychosocial Development

**DOI:** 10.3389/fendo.2019.00644

**Published:** 2019-09-19

**Authors:** Ahmet Uçar, Jarod Sze Choong Wong, Feyza Darendeliler, Jeff M. P. Holly, Derek Leroith

**Affiliations:** ^1^Pediatric Endocrinology and Diabetes Clinic, University of Health Sciences, Sişli Hamidiye Etfal Education and Research Hospital, Istanbul, Turkey; ^2^Developmental Endocrinology Research Group, Royal Hospital for Sick Children, University of Glasgow, Glasgow, United Kingdom; ^3^Department of Pediatric Endocrinology and Diabetes, Istanbul University, Istanbul, Turkey; ^4^Faculty of Medicine, School of Translational Health Science, University of Bristol, Southmead Hospital, Bristol, United Kingdom; ^5^Icahn School of Medicine at Mount Sinai, New York, NY, United States

**Keywords:** Turner syndrome, growth, puberty, psychosocial, cardiovascular risk

Turner syndrome (TS) is the most common female sex chromosome disorder with an incidence of 1 in 2,000 to 1 in 2,500 live female births (1). Individuals may be diagnosed at different stages of life beginning from *in utero* till adulthood, abnormal maternal screening, or fetal abnormalities, in infancy through the presence of lymphedema, in childhood as a result of growth failure, in adolescence as a result of short stature with pubertal delay, and in adulthood as a result of premature ovarian failure (2). The key to the care of this population includes proactive screening for co-existing medical conditions, including imaging for cardiac and renal anomalies, and monitoring for obesity and hypertension, developmental/psychoeducational abnormalities, hearing loss, autoimmune diseases, and short stature. Ovarian dysfunction and infertility should be anticipated (2).

The purpose of this Research Topic is to gather together research and review papers, which may serve to highlight the diverse challenges in the care of females with TS with the expectation that this will allow more critical appraisal of existing studies, identify critical research gaps, and pave the path for future studies.

In terms of addressing issues regarding growth and puberty in females with TS, Gawlik et al. document successful induction and progression of puberty by transdermal estrogen in girls with TS at a mean age of 15.1 year over a mean follow-up period of 2.4 year. They also report adequate increase in uterine size without compromising stature, irrespective of the karyotype status. Therefore, the fixed-dose transdermal estrogen regimen suggested by the authors seems to be effective in females with delayed diagnosis of TS. Regarding the growth and pubertal timing in females with TS, Woelfle et al. present a comprehensive report based on KIGS® (Pfizer International Growth Database including 7,219 females with TS between 1987 and 2012), and they demonstrate evidence of positive secular trends on age at onset of puberty and on final height akin to that reported on the normal population. These finding may be indicative of earlier diagnosis and thus, of earlier start of GH and estrogen treatments than in the past. Because KIGS® data exclusively include GH-treated females with TS, whether these trends also apply to growth hormone (GH)-naïve counterparts is currently unknown. However, the doubling in prevalence in spontaneous puberty does suggest that environment-related trends may also apply to females with TS.

In addition to induction of puberty, the bone-health related advantages of estrogen replacement in TS have been longitudinally evaluated by Li et al. using a regional estrogen replacement protocol in China, and the authors report the positive impact of estrogen on bone mineral density and muscle strength despite a relatively short term follow-up period.

It is well-established that females with TS are at increased risk of excess adiposity and its related complications. The tempo of the derangement in metabolic health profile and the associated key factors have not been adequately studied. To this end, Lebenthal et al. document abnormal metabolic profiles in young prepubertal girls with TS, which confirms the presence of risk factors inherent to TS itself. The time-related increase in metabolic derangements with an increase in prevalence of overweight/obesity status also confirm that non-TS related factors as in the general population are also operative in TS. While the more prominent clustering of metabolic anomalies in females with 45,XO karyotype may suggest closer follow-up and earlier intervention in this group, factors associated with a more dismal metabolic outcome in 45,XO females await further studies. In a theme parallel with this latter article, Sun et al. review derangements in pancreatic β- cell function and their reflections on glucose metabolism in TS. The β- cell failure in TS may be due to Xp hapotype gene deficiency and to overexpression of some genes of Xq; this is also an area that also awaits further studies. Although females with TS are well-established to have increased autoimmunity, its association with dysglycemia is currently unestablished. In their review, Sun et al. also indicate that the theoretical adverse effect of GH therapy on glycemic regulation in this non-GH deficient population has not been proven, possibly owing to increase in lean mass with GH treatment.

In a preliminary report on autoimmunity in TS, Gawlik et al. find no significant difference between females with TS and healthy controls regarding regulatory T cell percents, but in a subgroup analysis between anti- thyroid peroxidase antibody positive and negative females with TS, a trend toward iXq karyotype with reduced percents of helper T cells was observed. These findings call for further studies to reach hard end-point conclusions on the mechanisms of autoimmunity in TS.

Thoracic aortic disease, be it congenital or acquired, is a major determinant of morbidity and mortality in TS [reviewed in Mortensen et al. (3)]. Cardiovascular risk assessment in TS, particularly for aortic dissection, unfortunately has remained inadequate, which is due to a limited understanding of the pathophysiology of thoracic aortic disease in TS. Cardiovascular magnetic resonance (CVMR) is the gold standard for non-invasive assessment of thoracic aortic disease. Obara-Moszynska et al. confirm the superiority of CVMR over echocardiography in identifying anomalies such as dilatation of the aorta, pericardial fluid, and functional impairment of ventricles in a young females with TS. Aortic dissection has been reported as early as 4 year in TS (4). However, the availability of CVMR is limited in many developing countries, and it requires general anesthesia in patients under 6 years of age. These shortcomings of CVMR indicate the need to find potential markers to diagnose aortic pathology in TS. To this end, Mainkurve and O'Gorman reviewed the potential role of natriuretic peptides and osteoprogerin for aortic pathology in TS. While some associations of these markers have been found with aortic disease, their predictive value remains to be determined.

While the endocrine- related issues in TS are addressed in many studies, studies evaluating psychosocial problems in TS are scant [reviewed in Culen et al. (5)]. Referring to a former study of theirs (6) and the current study, Anaki et al. document that dysfunction in social tasks in TS is most likely due to spatial-visual factors, and that the capacity of females with TS to understand the emotional and cognitive status of others is similar to healthy controls.

Rovet and Van Vliet examine a subgroup of females with TS from the Canadian GH trial regarding potential psychosocial benefits of of GH treatment. Studies have shown that short children have been affected by juvenilization, teasing, bullying, victimization, loss of independence/ overprotection, and exclusion [reviewed in Lipman and McCurry (7)]. However, this conclusion has not been strongly confirmed by Rovet and Van Vliet, who document modest effect of GH treatment on psychosocial functioning in females with TS. In this study, Rovet and Van Vliet included a respectable number of patients with a relatively lower rate of follow-up data loss compared to other studies (8). Therefore, the authors conclude that it is important not to overemphasize the benefits of GH treatment on heightism since final height in many GH treated females with TS remains suboptimal.

In conclusion, we hope that this Research Topic will serve as a point of reference and source of inspiration for researchers and clinicians interested in addressing controversial issues related to the care of females with TS.

## Author Contributions

AU drafted the manuscript and agreed to be accountable for all aspects of the work in ensuring that questions related to the accuracy or integrity of any part of the work are appropriately investigated and resolved. All co-authors revised the manuscript for important intellectual content, and approved the final version to be published.

### Conflict of Interest Statement

The authors declare that the research was conducted in the absence of any commercial or financial relationships that could be construed as a potential conflict of interest.
